# Successful Orbital Atherectomy of Left Main Bifurcation Lesion Using Microcatheter Protection of Nonatherectomy Wire

**DOI:** 10.1016/j.jscai.2021.100007

**Published:** 2022-01-30

**Authors:** Alexander E. Sullivan, Julie Shelton, Colin Barker, Kashish Goel

**Affiliations:** Division of Cardiology, Vanderbilt University Medical Center, Nashville, TN

**Keywords:** Orbital atherectomy, Left main bifurcation, Calcified coronary artery disease

## Abstract

- Optimal PCI may be challenging with calcified left main bifurcation lesions
- Orbital atherectomy is a common strategy for lesion optimization
- Side branch occlusion is a feared complication of orbital atherectomy
- Utilizing microcatheter wire protection can minimize occlusion risk with orbital atherectomy

Atherectomy is a tool for calcium modification, but there is an increased risk of losing the side branch in left main (LM) bifurcation lesions. Protected atherectomy using dual-^1^ and single-guide catheter technique^2^ with mechanical support has been described as a strategy to minimize side branch closure and wire damage to nontarget vessel during bifurcation atherectomy. However, single-guide catheter technique has not been reported without the use of mechanical circulatory support.

We present a case of a 79-year-old man with severe, heavily calcified distal LM, proximal left anterior descending (LAD), and left circumflex artery (LCx) lesions (Fig. 1A, Supplemental Video 1) who was referred for complex percutaneous coronary intervention in the setting of reduced ejection fraction (40%), daily anginal symptoms, and viable myocardium on cardiac magnetic resonance imaging.

**Fig. 1:**
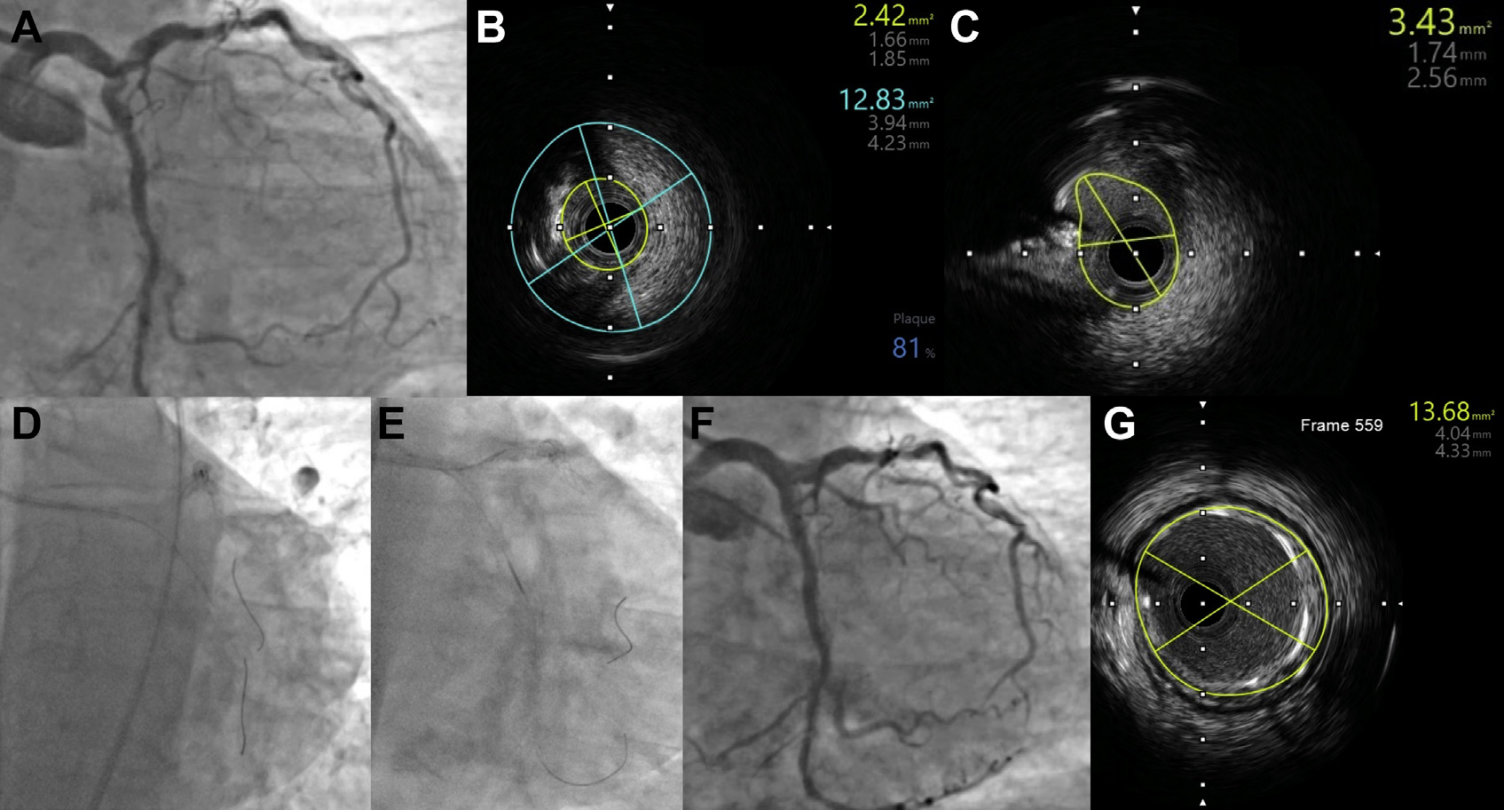
Orbital atherectomy of calcified left main bifurcation lesion. (A) Initial LM lesion, (B) initial LAD IVUS, (C) initial LCX IVUS, (D) OA of LM-LCX, (E) OA of LM-LAD, (F) final angiographic result, (G) final LM IVUS. IVUS, intravascular ultrasound; LAD, left anterior descending artery; LCX, left circumflex artery; LM, left main; OA, orbital atherectomy.

Peripheral angiography revealed severe peripheral vascular disease prohibitive for mechanical support devices. An 8-F sheath was placed in the left femoral artery, and an 8-F XB 4.0 guide catheter was used. Intravascular ultrasound (IVUS) confirmed severe calcified stenoses in LM, LAD, and LCx (Fig. 1B, C, Supplemental Videos 2 and 3). After placement of ViperWire Advance with Flex Tip (Cardiovascular Systems Inc) in the LM-LCx for orbital atherectomy, a Runthrough wire (Terumo Interventional Systems) was placed in the LAD “protected” with a Turnpike LP microcatheter (Teleflex). Orbital atherectomy (2 runs) of the distal LM and proximal circumflex arteries was performed at low speed (Fig. 1D). The microcatheter was advanced after each pass to minimize damage. During wire exchange between the LAD and LCx, the microcatheter was removed, flushed, and checked for any damage. It was then used to “protect” the LM-LCx wire during 4 total atherectomy passes from LM into the LAD at both high and low speeds (Fig. 1E, Supplemental Video 4). After atherectomy of each vessel, the microcatheter was examined, and minor abrasions similar to those reported by Panchal et al. were noted on the catheter without any significant catheter injury.^2^ Therefore, the same microcatheter was used during atherectomy of both the LAD and LCx. IVUS-guided percutaneous coronary intervention using double kissing crush technique was performed with the placement of a 3.5 ​× ​18-mm drug-eluting stent in the LCx-LM and a 4.0 ​× ​18-mm drug-eluting stent in the LM-LAD with postdilation of the LM using a 5.0-mm noncompliant balloon. Final angiography and IVUS showed excellent expansion and apposition (Fig. 1F, G, Supplemental Video 5). At 3-month follow-up, the patient was doing well with resolution of anginal symptoms and normalization of left ventricular ejection fraction.

Microcatheter-“protected” orbital atherectomy of 10.13039/100006186LM bifurcation lesions can be successfully performed using a single 8-F guide without the need for mechanical circulatory support in patients with prohibitive comorbidities and peripheral arterial disease.
